# Buffering the Fear of COVID-19: Social Connectedness Mediates the Relationship between Fear of COVID-19 and Psychological Wellbeing

**DOI:** 10.3390/bs12030086

**Published:** 2022-03-21

**Authors:** Ashley Humphrey, Evita March, Andrew P. Lavender, Kyle J. Miller, Marlies Alvarenga, Christopher Mesagno

**Affiliations:** 1 School of Science, Psychology and Sport, Federation University, Ballarat, VIC 3353, Australia; e.march@federation.edu.au (E.M.); a.lavender@federation.edu.au (A.P.L.); kj.miller@federation.edu.au (K.J.M.); m.alvarenga@federation.edu.au (M.A.); chris.mesagno@vu.edu.au (C.M.); 2 Institute of Health and Sport, Victoria University, Melbourne, VIC 3000, Australia

**Keywords:** COVID-19, social connectedness, depression, stress, resilience

## Abstract

Social connections are crucial for an individual’s health, wellbeing, and overall effective functioning. During the COVID-19 pandemic, one major preventative effort for reducing the spread of COVID-19 involved restricting people’s typical social interactions through physical distancing and isolation. The current cross-sectional study, conducted during the COVID-19 pandemic, explored the relationship among fear of COVID-19, social connectedness, resilience, depressive symptomologies, and self-perceived stress. Participants (*N* = 174) completed an anonymous, online questionnaire, and results indicated that social connectedness mediated the relationship between fear of COVID-19 and psychological wellbeing. In contrast, the relationship between fear of COVID-19 and psychological wellbeing was not mediated by resilience. These findings highlight the important role that social connections and resilience play in buffering against negative psychological wellbeing outcomes, especially during a pandemic.

## 1. Introduction

A strong level of social connectedness—defined as the experience of feeling close and connected to others—promotes lower rates of anxiety and depression, higher self-esteem, and improved health outcomes for general populations [1]. Conversely, social isolation—the state and experience of reduced social contact—can have serious negative consequences for our physical and mental health [2]. The benefits of social connectedness are particularly poignant during times of uncertainty and distress, when social contact can act as a buffer against adversity and suffering [3,4]. Indeed, the stress-buffering hypothesis holds that the effect of stress is weaker among those with high levels of social support [5]. Since early 2020 when the World Health Organisation declared the coronavirus virus (COVID-19) a pandemic, the COVID-19 pandemic triggered uncertainty and stress across the globe [6], posing an immediate threat to people’s health and wellbeing and leading to increased fear worldwide [7]. Indeed, fear of COVID-19 has been identified as one of the most frequent emotions associated with the pandemic. Worry, health-related anxiety, media exposure, and the risks for loved ones are amongst major predictors for fear of this disease [8,9]. This fear was often further exacerbated by required policy responses to COVID-19, whereby many governments worldwide implemented strong social distancing measures, such as increased physical distance between people, forced lockdowns (i.e., stay at home requirements), and measures of self-isolation. This challenging social environment raises the need to consider how the loss of social connections impact resilience and psychological wellbeing amidst this global pandemic [4,10,11].

Social connectedness is a strong positive predictor of resilience to stress following exposure to trauma and disasters [12]. Psychological resilience is the process of adapting well in the face of adversity, trauma, tragedy, threats, or significant sources of stress [13]. In addition to “bouncing back” from these difficult experiences, resilience can also involve profound personal growth by way of enhancing self-sufficiency and independence amidst challenging circumstances [14]. In the context of COVID-19, resilience may mitigate anxiety, depression, and COVID-19 related worries [15]. Although the COVID-19 pandemic has been linked to increased fear and stress [7], there is reason to expect that resilience might buffer the stress associated with COVID-19, subsequently reducing the impact of that stress on psychological wellbeing [11,16].

The emergence of COVID-19 and the subsequent measures aimed at preventing the spread of the virus has led to significant disruptions and restrictions in people’s lives. Most notably, these measures have significantly impacted social interactions, including (but not limited to) reductions in face-to-face meetings and limited physical contact with social support networks. Importantly, researchers have indicated that social interactions work as support and coping mechanisms for individuals to better deal with adverse events [17]. When considering that social restrictions may have exacerbated the negative impact of other factors associated with lockdown and restrictions, such as changes in income, employment, and parenting commitments [18], it could be argued that the true social impact of the COVID-19 pandemic might be greater and far reaching than what is currently recognised. However, the novel environment of COVID-19 provides an opportunistic setting to explore the role social connectedness has on psychological wellbeing. Exploration of whether social connectedness and resilience mediate psychological wellbeing will have significant implications not just for the current context of a pandemic, but for navigating stressful and impactful future situations where social connectedness is impacted.

In this study, we aim to investigate the relationships between social connectedness, resilience, fear of COVID-19, and psychological wellbeing (operationalised by depression and stress). Furthermore, we aim to explore the utility for social connectedness and resilience to indirectly buffer the impact of fear of COVID-19 on psychological wellbeing. It is hypothesised that there will be a significant, negative relationship between fear of COVID-19 and psychological wellbeing (H1). Furthermore, we hypothesise that there will be significant, positive relationships between social connectedness and psychological wellbeing (H2), and resilience and psychological wellbeing (H3). Lastly, we predict that social connectedness (H4) and resilience (H5) will each mediate the relationship between fear of COVID-19 and psychological wellbeing, buffering the negative impact.

## 2. Method

### 2.1. Participants and Procedure

A survey-based study was conducted with the aim of conducting a cross-sectional analysis. Participants were 174 English speaking individuals from the USA and Australia representing a random selection of the wider population and were aged between 19 and 80 years (*M_age_* = 39.06, *SD_age_* = 13.36). Participants were required to be over the age of 18 and English speaking. As a general sample was sought, there was no other exclusion criteria for participation. Participants were asked to report their biological sex (85 male, 88 female, 1 unidentified). Eight participants were excluded from the analysis because they did not have sufficient data for more than one outcome variable. Following University Human Research Ethics Committee approval, participants were recruited from May 2020 to June 2020 via snowballing techniques on social media (e.g., Facebook, Twitter, and Instagram posts), with the online questionnaire link embedded in the post. During this time, participants in both countries were enduring “stay at home” measures which heavily restricted people’s propensity to interact with others. As an incentive, participants were offered a chance to win one (of two) randomly drawn AU$50 e-gift vouchers. The questionnaire was part of a broader study examining some of the social impacts associated with COVID-19 and took approximately 30 min to complete.

An a priori calculation using G Power [19] with power set at 0.95, alpha at 0.05, effect size at 0.15, with 3 predictor variables (social connectedness, resilience and fear of COVID-19) indicated a minimum sample size of 119 was needed for sufficient power, which was satisfied with the current sample size of *N* = 174.

### 2.2. Measures

The online questionnaire included demographics (i.e., gender, age, country of residence) and the following measures.

**Revised Social Connectedness Scale (SCS-R)** [20]: The SCS-R consists of 10 items (e.g., “I feel understood by people I know”; current α = 0.91) to assess interpersonal connectedness. Participants respond to statements on a 6-point Likert scale (1 = *strongly disagree*; 6 = *strongly agree*). After reverse scoring, items are summed for a total score with higher scores reflecting higher social connectedness;

**Brief Resilience Scale (BRS)** [21]: The BRS comprises six items (e.g., “I tend to bounce back quickly after hard times”; current α = 0.82) that assess an individual’s level of resilience. Participants respond on a 5-point Likert scale (1 = *strongly disagree*; 5 = *strongly agree*). After reverse scoring, total scores are summed then divided by the number of items, with higher scores indicate greater resilience;

**Centre for Epidemiologic Studies Depression Scale (CES-D)** [22]: The CES-D is a 20-item measure that assesses depressive symptoms in the past week. Participants respond to items (e.g., “I felt depressed”; current α = 0.93) on a 4-point Likert scale (0 = *rarely or none of the time*; 3 = *most or all of the time*). After reverse scoring, items are summed with higher scores indicating greater feelings of depression;

**Perceived Stress Scale (PSS-10)** [23]: The PSS-10 is a 10-item measure that determines perceived stress over the past month. Participants respond to items (e.g., “In the last month, how often have you felt that you were unable to control the important things in your life?”; current α = 0.79) on a 5-point Likert scale (0 = *never*; 4 = *very often)*. After reverse scoring, items are summed with higher scores indicating higher perceived stress:

**Fear of COVID-19 Scale (FCV-19S)** [24]: The FCV-19S comprises 7 items (e.g., “I am most afraid of coronavirus-19”; current α = 0.92) to assess fear of COVID-19. Participants respond on a 5-point Likert scale (1 = *strongly disagree*; 5 = *strongly agree*). Total scores are summed with higher scores indicating greater fear of Coronavirus.

## 3. Results

Data was firstly uploaded to SPSS Version 26 for analysis and a series of correlation analyses were then run. The tests of general assumptions of linear regression showed all measures were normally distributed according to the tests of kurtosis and skewness and within acceptable bounds for parametric testing at *p* < 0.05. Zero-order correlations were firstly used to examine the initial relationships between the variables. Descriptive statistics and correlations are shown in Table 1. All variables demonstrated significant bivariate correlations.

### Path Analysis

**Figure 1:**
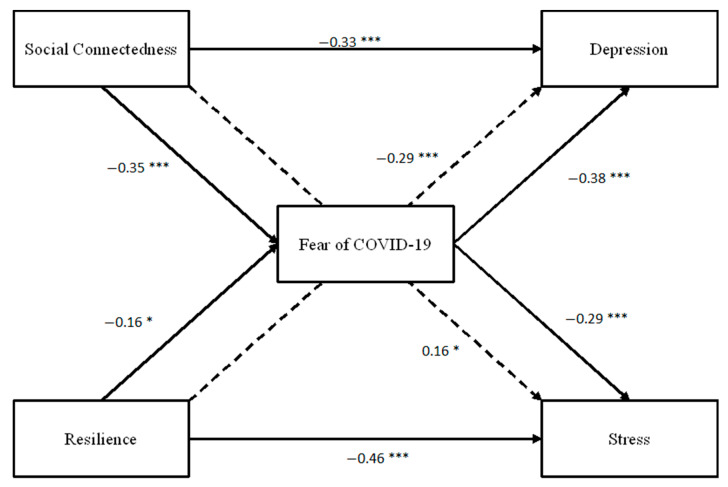
*Coefficients for path analysis model. Coefficients are standardized.* Note. *** *p* < 0.001, ** *p* < 0.01, * *p* < 0.05. Coefficients for the path analysis model. Coefficients are standardised. Dotted lines represent direct associations between (i) social connectedness and stress (*r* = 0.16), and (ii) resilience and depression (*r* = −0.29).

To examine the mediating role of social connectedness and resilience on the relationships between fear of COVID-19 and psychological wellbeing, a path analysis was performed via AMOS (see Figure 1).

Table 2 presents the standardised estimates of total, direct, and indirect effects on psychological wellbeing.

Examination of bias corrected bootstrap 95% confidence intervals indicated that, via Fear of COVID-19, there was a statistically significant indirect effect of social connectedness and depression (LCI = −0.18, UCI = −0.03, *p* = 0.011), and social connectedness and stress (LCI = −0.07, UCI = −0.01, *p* = 0.007). However, bias corrected bootstrap 95% CIs indicated that, via Fear of COVID-19, there was no statistically significant indirect effect of resilience and depression (LCI = −0.35, UCI = 0.05, *p* = 0.174), and no statistically significant indirect effect of resilience and stress (LCI = −0.14, UCI = 0.18, *p* = 0.182).

## 4. Discussion

In this study, we explored relations among social connectedness, resilience, fear of COVID-19, and psychological wellbeing (operationalised by depression and anxiety), and the utility for social connectedness and resilience to indirectly buffer the impact of fear of COVID-19 on psychological wellbeing. In line with our predictions and corroborating recent findings [8], our results revealed that there was a significant negative relationship between fear of COVID-19 and psychological wellbeing (H1), and significant positive relations between social connectedness (H2) and resilience (H3). We further hypothesised that social connectedness (H4) and resilience (H5) would mediate the relationship between fear of COVID-19 and psychological wellbeing. Our results partially supported these hypotheses, by showing social connectedness (but not resilience) positively mediated this relationship, corroborating the theoretical framework of the stress buffering hypothesis [5]. These results contribute to the extant literature on the importance of social connectedness [4,10] and resilience [15,16] for wellbeing amidst the COVID-19 pandemic. Taken together, the current findings highlight that social connectedness may play a unique role in mitigating distress during the COVID-19 pandemic, illustrating the importance of fostering and maintaining social connections, particularly during times of adversity, in order to reduce stress and anxiety [10].

### Limitations, Future Research, and Conclusions

These results are limited by the absence of pre-COVID baseline assessments when physical distancing measures were not in place. Thus, it could be argued that these findings only apply to the time-period when COVID-19 was newly emerging, and as such, most of the population was experiencing the obscurity of the pandemic and lockdowns. However, examining this association during this time of the crisis has allowed us to test the robustness of these experimental associations in a real-life situation, with these findings offering an important snapshot during a time of imposed social isolation and heightened stress. These findings are also limited by the correlational and cross-sectional design, limiting inferences about the causal direction of the relationships. It is of course possible that people who more often feel depressed or stressed could be more disconnected socially, less resilient, and more fearful of COVID-19. Future research could endeavour to explore these mediators via longitudinal design, thus permitting causal inferences.

The current study aimed to explore the role social connectedness may play in buffering against negative physical and mental health outcomes during difficult and uncertain times. The results of the current study highlight the importance of maintaining adequate social connections during times of uncertainty and stress, to buffer against the negative effects of highly challenging life events. With people living in developed countries shown to be increasingly individualistic in their social orientations, such results are perhaps more important than ever [25]. Despite the current study being contextualised in the novel context of the COVID-19 pandemic, we posit these results can be extrapolated to highlight the critical role social connectedness and resilience (to a lesser extent, based on our findings) play in a range of stressful life events.

## Figures and Tables

**Table 1:** Descriptive statistics and bivariate correlations.

	*M*	*SD*	2.	3.	4.	5.
1. Social connectedness	77.20	17.01	0.55 ***	−0.60 ***	−0.50 ***	−0.43 ***
2. Resilience	19.37	5.06		−0.57 ***	−0.59 ***	−0.34 ***
3. Depression	21.94	13.28			0.63 ***	0.58 ***
4. Perceived stress	19.23	6.46				0.49 ***
5. Fear of COVID-19	21.18	7.28				

*Note:* *** *p* < 0.001, ** *p* < 0.01, * *p* < 0.05.

**Table 2:** Direct and indirect effects on psychological wellbeing.

	Pathway	Standardised Effect
Social Connectedness	→ Fear of COVID-19 (total effect)	−0.35
	→ Fear of COVID-19 (direct effect)	−0.16
	→ Depression (total effect)	−0.33
	→ Depression (direct effect)	−0.29
	→ Depression (total indirect effect)	−0.14
	→ Stress (total effect)	−0.16
	→ Stress (direct effect)	0.32
	→ Stress (total indirect effect)	−0.10
Resilience	→ Fear of COVID-19 (total effect)	−0.16
	→ Fear of COVID-19 (direct effect)	−0.16
	→ Depression (total effect)	−0.35
	→ Depression (direct effect)	−0.29
	→ Depression (total indirect effect)	−0.06
	→ Stress (total effect)	−0.50
	→ Stress (direct effect)	−0.46
	→ Stress (total indirect effect)	−0.05

## Data Availability

The data that support the findings of this study are available on request from the corresponding author.
